# Lifestyle and SSRI Interventions in Pediatric Cyclic Vomiting Syndrome: Rethinking First-Line Management

**DOI:** 10.3390/children12080964

**Published:** 2025-07-23

**Authors:** Cansu Altuntaş, Doğa Sevinçok, Merve Hilal Dolu, Ece Gültekin

**Affiliations:** 1 Pediatric Gastroenterology, Medical Faculty, Istinye University, Istanbul 34510, Türkiye; 2 Child and Adolescent Psychiatry, Medical Faculty, Istinye University, Istanbul 34510, Türkiye; doga.sevincok@istinye.edu.tr; 3 Pediatric Neurology, Medical Faculty, Istinye University, Istanbul 34510, Türkiye; mervehilal.dolu@isu.edu.tr; 4 Pediatric Neurology, Medical Faculty, Biruni University, Istanbul 34010, Türkiye; egultekin@biruni.edu.tr

**Keywords:** cyclic vomiting syndrome, pediatric, selective serotonin reuptake inhibitors, cyproheptadine, lifestyle modification, anxiety, ROME IV criteria

## Abstract

**Background**: Cyclic vomiting syndrome (CVS) is a functional gastrointestinal disorder characterized by recurrent episodes of intense nausea and vomiting. Despite increasing awareness, a standardized treatment approach remains lacking in pediatric populations. Lifestyle factors and anxiety are common triggers, yet their systematic management has not been fully incorporated into therapeutic strategies. **Objective**: To evaluate the effectiveness of lifestyle modifications and selective serotonin reuptake inhibitors (SSRIs) in the management of pediatric CVS and to compare their outcomes with standard cyproheptadine prophylaxis. **Methods**: This retrospective study included 119 patients aged 1.2–17.5 years who were diagnosed with CVS according to Rome IV criteria between September 2021 and January 2025. Clinical, psychiatric, and lifestyle data were retrieved from the university’s digital medical records. Patients were grouped according to treatment modality: cyproheptadine, SSRI, or acute attack management alone. Treatment success at 12 weeks was defined as complete cessation of vomiting episodes or absence of hospitalization, prolonged attacks, and school/work absenteeism. **Results**: Anxiety symptoms were present in 78.2% of patients. SSRIs were prescribed to 34 patients with moderate to severe anxiety, all of whom achieved treatment success. Lifestyle adherence was observed in 73.9% and was found to be a predictor of treatment success. Cyproheptadine was administered to 66 patients but did not provide additional benefit over effective lifestyle modification. Six patients discontinued cyproheptadine due to drowsiness or weight gain. **Conclusions**: Lifestyle interventions significantly improve outcomes in pediatric CVS. SSRIs represent a safe and effective prophylactic option for patients with comorbid anxiety or poor adherence to behavioral recommendations. These findings support the integration of psychosocial and lifestyle-based strategies into standard CVS treatment protocols.

## 1. Introduction

Cyclic vomiting syndrome (CVS) is a functional gastrointestinal disorder that may begin during infancy and can significantly impair quality of life throughout childhood. Affected patients typically experience prodromal symptoms—such as fatigue, dysphoria, and pallor—followed by episodes of intense retching. These vomiting attacks may continue even after gastric emptying and are often accompanied by abdominal pain and diarrhea. Severe and prolonged attacks frequently result in emergency department visits. Recurrent episodes can lead to school absenteeism in children and work loss among caregivers, creating notable social and economic burdens [1].

Diagnostic evaluations performed during asymptomatic periods usually yield normal results, apart from dehydration-related abnormalities observed during attacks. Clinical suspicion remains key in establishing the diagnosis, and the Rome IV criteria—currently the standard diagnostic framework for functional gastrointestinal disorders—are used to guide the diagnosis of CVS [2]. Due to limited awareness of the disease, clinical suspicion is often delayed, resulting in late diagnosis and postponed initiation of appropriate treatment. The absence of a fully understood etiopathogenesis and the high prevalence of comorbid conditions have hindered the establishment of standardized treatment protocols. Owing to its similarities with migraine syndromes, CVS has been found to share common triggering factors such as inadequate sleep, unhealthy diet, dehydration, and prolonged screen time [3,4]. In recent years, increasing attention has been directed toward the psychosocial contributors to CVS, with anxiety now recognized as a significant precipitating factor [1,5]. However, the impact of effectively managing these psychiatric and lifestyle-related issues on treatment outcomes has not been fully explored.

The aim of this study was to evaluate the impact of lifestyle modifications and treatment outcomes on patients diagnosed with cyclic vomiting syndrome according to the Rome IV criteria. The following lifestyle and psychosocial risk factors were assessed as part of the lifestyle modification evaluation:

- Unhealthy dietary habits, including
  ○ Frequent consumption of junk food (defined as more than twice per week),
  ○ Skipping meals,
  ○ Meals inconsistent with a healthy Mediterranean-style diet,
  ○ Inadequate hydration
- Poor sleep hygiene, defined as failure to achieve a minimum of 8 h of regular nighttime sleep
- Excessive screen time, defined as more than 2 h per day outside of school hours
- Presence of anxiety symptoms, as reported by the patient, family, or school staff

## 2. Materials and Methods

This retrospective single-center study included patients aged 1 to 18 years who were admitted to the pediatric gastroenterology outpatient clinic between September 2021 and January 2025 and met the diagnostic criteria for cyclic vomiting syndrome (CVS) according to the Rome IV criteria (Table 1) [2].

As part of the diagnostic work-up, all patients underwent the following investigations, which yielded normal results:

- Laboratory tests: Kidney function tests, liver transaminases, C-reactive protein, erythrocyte sedimentation rate, serum amyloid A, fibrinogen, celiac screening (tissue transglutaminase and/or endomysial antibodies), food allergy tests (when clinically indicated), urinary organic acid analysis, and metabolic screening with tandem mass spectrometry.
- Radiological tests: Abdominal ultrasonography and abdominal X-ray performed both during and between attacks.
- Upper gastrointestinal endoscopy: Performed with biopsies, which revealed no abnormalities.

As part of the diagnostic evaluation, all patients were assessed by both the pediatric neurology and child psychiatry departments. Patients with abnormal cranial computed tomography (CT) or magnetic resonance imaging (MRI) findings, abnormal electroencephalogram (EEG) results, or any metabolic or genetic disorder that could account for vomiting episodes other than CVS were excluded. Treatment response was reassessed at the 12th week of therapy. Ethical approval was obtained from the Istinye University Ethical Board on 28 March 2025 (approval number: 25–45).

### 2.1. Treatment Protocol

**Figure 1:**
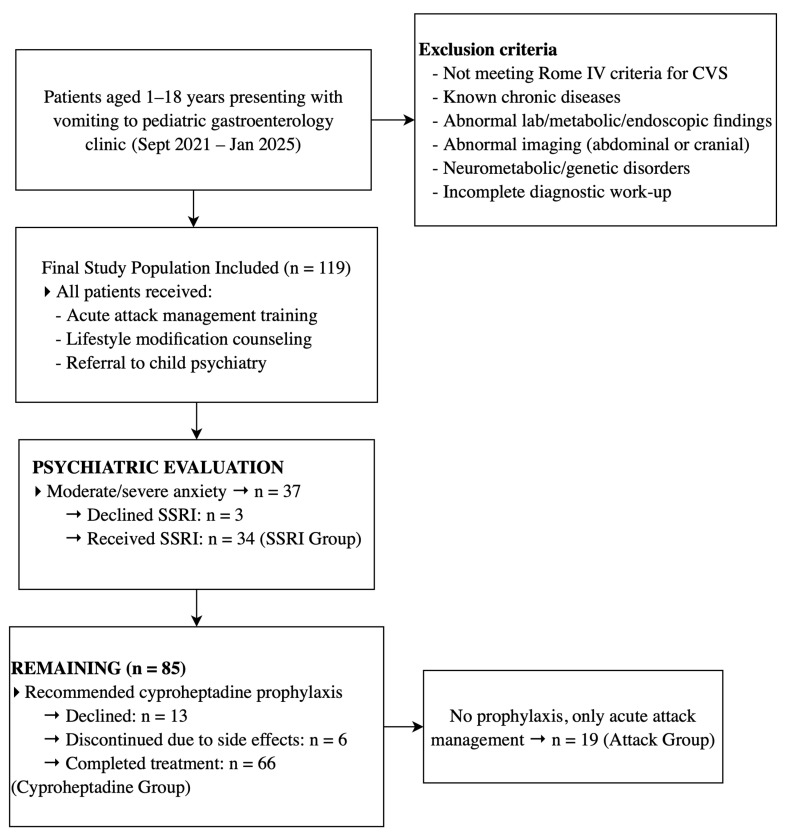
Flowchart of the study design and patient distribution across treatment groups.; CVS: cyclic vomiting syndrome; SSRI: selective serotonin reuptake inhibitor.

Each patient diagnosed with CVS was evaluated by the pediatric gastroenterology, pediatric neurology, and child psychiatry departments, and treatment decisions were made through multidisciplinary consensus. The treatment protocol is outlined as a flowchart in Figure 1.

The treatment protocol at our center consisted of the following components:

- Acute Attack Management [6]:

Upon recognition of a typical attack, patients received sublingual ondansetron at a dose of 0.15 mg/kg, followed 10 minutes later by a non-steroidal anti-inflammatory drug (NSAID), preferably ibuprofen (10–15 mg/kg, maximum dose 400 mg). Patients were then advised to rest in a quiet, dark room and were supported with oral hydration when possible. All patients were provided with a written copy of the acute attack management protocol, applicable both for home-based care and, when necessary, for emergency department management involving intravenous treatment and benzodiazepines for refractory cases.

- Lifestyle Modifications:

All patients received structured counseling focused on [7]

○ Maintaining regular meal patterns with three main meals consistent with a healthy Mediterranean-style diet, supplemented by 1–2 fruit-based snacks per day,
○ Ensuring adequate hydration appropriate for age and body weight,
○ Achieving at least 8 h of nighttime sleep in a dark, quiet environment (sleep hygiene),
○ Limiting screen time to fewer than 2 h per day (outside of school hours),
○ Adhering to psychiatric treatment and follow-up recommendations.

- Prophylaxis with Cyproheptadine [6]:

Patients were recommended to receive cyproheptadine at a dose of 0.25–0.3 mg/kg/day (maximum 4 mg), typically administered in the evening before bedtime.

- Treatment Based on Psychiatric Indication [8]:

Patients determined by psychiatric evaluation to require SSRI therapy were included in the SSRI treatment group. If these patients had previously received cyproheptadine, it was discontinued upon initiation of SSRI treatment.

The choice of SSRI and dosage was determined by psychiatrist and included

○ Sertraline, initiated at 12.5–25 mg/day in the first week and increased to 50 mg/day thereafter,
○ Fluoxetine, initiated at 5–10 mg/day, titrated up to a maximum of 20 mg/day.

All patients were instructed to promptly report any adverse effects or discontinuation of treatment. Patients who declined or were unable to tolerate prophylactic therapy remained in the study under the acute attack management group, provided they agreed to regular clinical follow-up. All clinical and demographic data, including attack characteristics, hospitalization history, lifestyle factors, and treatment details, were retrospectively retrieved from the institutional electronic medical record system.

### 2.2. 12-Week Follow-Up and Lifestyle Adherence Assessment

At the 12th-week follow-up visit, both the patient and caregiver were interviewed regarding adherence to lifestyle recommendations. Effective lifestyle modification was defined as meeting all of the following:

- No skipped meals; three regular and nutritious main meals consistent with the Mediterranean diet, plus 1–2 fruit-based snacks daily,
- Adequate hydration tailored to the child’s age and weight,
- At least 8 h of nighttime sleep in a dark environment (good sleep hygiene),
- Screen time limited to ≤2 h per day (outside school hours),
- Full compliance with psychiatric department recommendations for therapy or follow-up.

Additionally, the presence of CVS attacks, attack frequency and duration, hospitalization requirements, school absenteeism for the child, and work absenteeism for the caregiver were recorded.

### 2.3. Definition of Treatment Outcomes

Treatment success was defined as meeting either the first criterion only or the second and third together:

- Complete cessation of vomiting attacks,
- No attacks requiring hospitalization,
- No school or work absenteeism due to attacks.

Treatment failure was defined by the presence of one or more of the following:

- Attacks requiring hospitalization,
- No reduction or an increase in attack frequency or intensity,
- Attacks resulting in school absenteeism for the child or work absenteeism for the caregiver,
- Any single attack lasting longer than 24 h.

### 2.4. Statistical Analysis

Data analysis was completed with the IBM SPSS Statistics ver. 25 (IBM Corporation, Armonk, NY, USA) package program. Categorical data were expressed as numbers (n) and percentage (%), while quantitative data were given as mean ± SD or median (min–max); where appropriate. The Kolmogorov–Smirnov and Levene tests were used to investigate whether the normal distribution and homogeneity of variance assumptions was met. For comparisons between two independent groups, normally distributed variables were evaluated with the Student’s *t*-test, whereas non-normal variables were evaluated with the Mann–Whitney U test. When the number of independent groups exceeded two, an omnibus test was first conducted: a one-way ANOVA for continuous variables when parametric test assumptions were met, or the Kruskal–Wallis test when these assumptions were violated.. If these variance analyses revealed a significant overall difference, the specific group pairs responsible for the difference were identified via Tukey’s HSD test (following ANOVA) or the Dunn Bonferroni post-hoc test (following Kruskal–Wallis). Unless otherwise stated, categorical variables were compared with Pearson’s χ^2^ test. In 2 × 2 contingency tables, Fisher’s exact test was used when the expected frequency was less than 5 in at least ¼ of the cells in 2 × 2 crosstabs, and the χ^2^ test with continuity correction was used when the expected frequency was between 5 and 25. In R × C tables (where at least one row or column variable had >2 categories), the Fisher–Freeman–Halton test was employed when the expected frequency in ¼ of cells was <5. The statistical significance of the results was determined by a *p*-value of 0.05.

## 3. Results

Between September 2021 and January 2025, a total of 123 patients were identified as meeting the Rome IV diagnostic criteria for CVS in the pediatric gastroenterology outpatient clinic. During the study period, five patients were excluded due to alternative diagnoses: three due to abnormal EEG findings, one due to a diagnosis of Fragile X syndrome, and one due to mitochondrial encephalomyopathy with lactic acidosis and stroke-like episodes (MELAS). Ultimately, data from 119 patients aged between 1.2 and 17.5 years were included in the final analysis. The study flowchart and distribution of treatment groups are summarized in Figure 1.

The mean age of the patients was 8.7 ± 3.9 years. Of the 119 patients, 61 (51.3%) were female and 58 (48.7%) were male. The mean BMI SDS was −0.15 ± 1.17. The median number of vomiting attacks prior to diagnosis was five (range: 2–15). A total of 62 patients (52.1%) had a history of hospitalization due to CVS attacks. The demographic characteristics of all included patients are summarized in Table 2.

**Figure 2:**
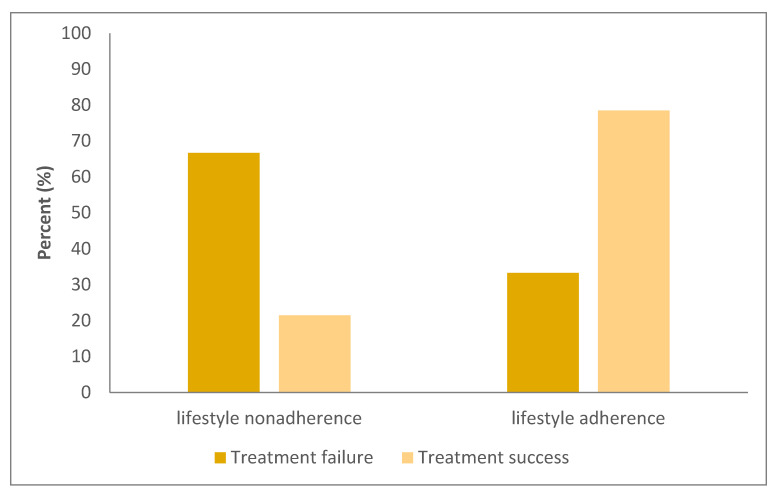
Treatment success and failure rates between lifestyle adherence and nonadherence groups (*p* = 0.002).

When treatment success was evaluated across the entire cohort, lifestyle adherence was found to be significantly associated with improved outcomes (*p* = 0.002; Table 3, Figure 2). Additionally, the SSRI group demonstrated significantly higher treatment success compared to the attack and cyproheptadine groups (*p* = 0.018; Table 3). Table 3 summarizes the demographic features and treatment outcomes of the three treatment groups: attack, cyproheptadine, and SSRI. Patients in the SSRI group were older and had more frequent and prolonged attacks, a higher rate of hospitalization, and lower lifestyle adherence compared to the other groups. Despite these more severe clinical characteristics, treatment success was significantly higher in the SSRI group (Table 4).

Although the cyproheptadine group showed a treatment success rate of 86.4%, this was not significantly higher than that of the attack group when all patients were considered (Table 4). Among patients with poor lifestyle adherence, the addition of cyproheptadine did not result in significantly better outcomes compared to those who received only acute attack management (*p* = 0.2; Table 5).

All patients in the SSRI group achieved treatment success. While SSRI therapy did not show additional benefit over the attack group with high lifestyle adherence, it was significantly more effective in those who were unable to follow lifestyle recommendations, compared to both the cyproheptadine and attack groups (*p* = 0.013 and *p* < 0.001, respectively; Table 5).

## 4. Discussion

Our study is the first to systematically examine the direct impact of structured lifestyle modifications on disease progression and treatment outcomes in pediatric CVS. While known triggers—such as fasting, sleep deprivation, over-excitement, and unhealthy diet—have long been incorporated into CVS management [7], our findings show that adherence to structured behavioral recommendations significantly improved clinical outcomes. Patient education on sleep hygiene, hydration, nutrition, screen time reduction, and stress management played a key role in treatment success across all groups (Table 3, Figure 2). Notably, even patients who received no prophylactic treatment achieved comparable success when adhering to lifestyle modifications (Table 5). However, due to the non-homogeneous distribution of treatment groups—such as lower baseline attack frequency in the attack group and higher mean age in the SSRI group—these findings should not be interpreted as evidence that pharmacological treatments are unnecessary. Rather, they highlight the critical role of lifestyle interventions, which should complement medical therapy and be prioritized in treatment planning.

Although numerous review articles have proposed pharmacological treatment protocols for cyclic vomiting syndrome (CVS), a standardized treatment algorithm was only recently published by NASPGHAN in April 2025. This guideline also emphasizes the heterogeneity of current treatment approaches and highlights the need for standardized clinical data [6]. One of the major obstacles to developing uniform treatment protocols is the variable clinical presentation of the disease and the fact that the exact etiopathogenesis of CVS remains poorly understood. Several mechanisms have been proposed, including mitochondrial dysfunction, autonomic dysregulation, hypothalamic–pituitary–adrenal axis hyperreactivity, endocrine and endocannabinoid system imbalances, and genetic abnormalities [7,9,10]. In addition to these biological mechanisms, the high comorbidity with anxiety and the clinical overlap with migraine support provide growing evidence that lifestyle patterns and psychological well-being play a critical role in the pathophysiology and course of CVS [7,11,12]. As with many chronic conditions, recent studies recommend that non-pharmacological strategies—particularly lifestyle modifications and structured psychiatric evaluations—be implemented alongside pharmacological treatment to optimize outcomes [6,7,11,12,13].

Cyproheptadine is widely used as a prophylactic treatment for CVS, though previous studies have reported a broad dosing range and varied regimens involving two to three doses per day [7,13]. In patients who showed limited benefit from standard dosing, dose escalation up to 0.5 mg/kg/day or the addition of a daytime dose was attempted. However, these regimens could not be sustained due to excessive somnolence and were therefore excluded from our final treatment protocol. This finding underscores the need for caution regarding adverse effects when using cyproheptadine prophylactically. Notably, even at lower doses (0.25–0.3 mg/kg/day, single evening dose), 6 out of 72 patients discontinued treatment within the first 30 days due to excessive somnolence or increased appetite. A systematic review by Falsaperla et al. reported a nonresponse rate of approximately 24%, with weight gain and drowsiness being the most common side effects. In a separate study, the same authors found complete remission in only 21% of patients, while 43% showed no therapeutic response [14]. In our study, despite the side effects, the cyproheptadine treatment failure rate among those who completed follow-up was relatively low (13.6%). However, our data showed that cyproheptadine did not offer additional benefit over effective lifestyle adherence (Table 5). Therefore, we propose that lifestyle interventions should be prioritized in all CVS patients, and that cyproheptadine prophylaxis can be safely tapered or discontinued early in patients who achieve satisfactory symptom control through lifestyle modification alone, in order to avoid unnecessary adverse effects such as sedation and increased appetite.

A high rate of anxiety (78.2%) was observed in our study cohort. The association between CVS and anxiety is well established, with anxiety often considered a precipitating factor in CVS attacks [7,13]. Previous studies have reported increased hospitalization rates in CVS patients with comorbid anxiety and have even suggested that severe anxiety episodes may directly trigger CVS attacks [7,15]. In this context, the use of SSRIs has been proposed, particularly for patients with severe anxiety [13]. Despite these recommendations, most clinical studies to date have focused on the use of tricyclic antidepressants (TCAs), which have been incorporated into treatment protocols primarily for refractory CVS cases [6,7]. However, in Türkiye, TCAs are considered second-line therapy in pediatric anxiety due to their potential cardiac side effects. As a result, TCAs are generally not used for prophylaxis in CVS unless psychiatric symptoms persist despite SSRI therapy [8]. Although anxiety symptoms were present in 78.2% of our patients, not all were initiated on SSRIs. The decision to prescribe SSRIs was made by a child psychiatrist based on a comprehensive assessment of both symptom severity and the degree of functional impairment. In cases of mild, transient, or situational anxiety, non-pharmacological approaches such as brief psychiatric interventions were considered sufficient. SSRI treatment was recommended for 37 patients with moderate to severe anxiety, though 3 families declined the medication. These patients were instead assigned to the cyproheptadine group, and 34 patients ultimately completed the study in the SSRI treatment group. All patients receiving SSRI therapy had moderate to severe anxiety and presented with more frequent and prolonged attacks and higher rates of hospitalization compared to other groups, as previous studies also suggested (Table 4) [7,15]. Despite their more severe baseline, no hospitalization-requiring attacks occurred in the SSRI group after the second week of treatment. Patients in the SSRI group were older than those in other groups, likely due to the higher prevalence of anxiety during adolescence, and also showed lower rates of lifestyle adherence. The continued benefit of SSRI therapy, even in patients with poor lifestyle adherence, suggests that SSRIs may be particularly helpful for those unable to implement behavioral changes in the short term. This may help prevent hospitalizations and reduce school absenteeism, which is especially relevant in this age group.

To our knowledge, this is the first study to investigate the effects of SSRI therapy in patients with CVS. Although anxiety is highly prevalent in CVS, SSRIs are not commonly used due to concerns about suicidality and potential gastrointestinal side effects. In a 2017 study, Zar-Kessler et al. compared SSRIs and tricyclic antidepressants (TCAs) in children with functional abdominal pain and found that SSRIs were more effective (75% vs. 61%, *p* = 0.03) [16]. The authors concluded that SSRIs can be safely used by pediatric gastroenterologists with appropriate monitoring. Both drug classes had similar rates of gastrointestinal side effects, which occasionally led to treatment discontinuation. A recent placebo-controlled trial by Baumel et al. (2025) [17] showed that escitalopram improved nausea symptoms by the end of the first week, with no significant difference in overall side effects compared to placebo. Most side effects were mild and temporary. The authors suggested that SSRIs may help regulate the gut–brain axis by reducing anxiety and modulating serotonin pathways [17]. Similarly, Strawn et al. reported that sertraline reduced nausea in children with anxiety, without worsening symptoms like bloating, diarrhea, or constipation, over a 12-week period [18]. In our study, 8 of the 34 patients on SSRIs experienced mild nausea between episodes, possibly related to medication use, but none dis-continued treatment. Taken together with our results, these findings suggest that SSRIs can be a safe option for managing CVS when patients and families are properly informed about potential side effects. However, as shown in our study, SSRIs did not offer additional benefit compared to lifestyle modification. Therefore, in patients without psychiatric comorbidities, lifestyle interventions should remain the first-line treatment strategy.

In our study, all patients in the SSRI group had moderate to severe anxiety, distinguishing them from the other treatment groups. Because the treatment groups were not randomized and had different clinical features, we did not compare the success of SSRI and cyproheptadine directly. However, two important findings should be noted: patients who received SSRI treatment despite having more severe symptoms showed good treatment results, and patients in all groups who followed the lifestyle recommendations had better outcomes.

The main limitations of this study are that it was retrospective, the follow-up period was short (12 weeks), and we used caregiver reports to measure how well patients followed lifestyle advice and how severe their symptoms were when not hospitalized. Also, the treatment groups were not randomized, and SSRI treatment was given based on the psychiatrist’s decision, which may have caused some bias. Even with these limitations, we believe that this study shows how important lifestyle changes are for treatment success in CVS. It also suggests that SSRIs may be helpful for selected patients, especially those with anxiety or psychiatric comorbidities. We hope these findings will help guide future studies that include randomized, placebo-controlled trials and improve treatment for children with CVS.

## 5. Conclusions

This study demonstrates that lifestyle modifications play a key role in the treatment of pediatric cyclic vomiting syndrome. In addition, our findings suggest that SSRIs can be a safe and effective treatment option, especially for patients with coexisting anxiety. Further prospective studies are needed to confirm these results and to establish treatment approaches that combine lifestyle changes and mental health support in routine care.

## Figures and Tables

**Table 1:** Rome IV criteria for cyclic vomiting syndrome [2].

1- Two or more periods of intense, unremitting nausea and paroxysmal vomiting lasting for hours to days within a 6-month period.
2- Stereotypical pattern of episodes in each patient.
3- Episodes separated by weeks to months, with a return to baseline health between attacks.
4- Symptoms that cannot be attributed to another medical condition following appropriate evaluation.

**Table 2:** Demographic and clinical characteristics of the cases.

	n = 119
Age (years) *	8.7 ± 3.9
Range of age (years)	1.2–17.5
**Gender**	
Female	61 (51.3%)
Male	58 (48.7%)
Number of attacks per 6 months **	5 (2–15)
Duration of attacks (days) **	1 (1–8)
**Hospitalization**	62 (52.1%)
**Potential triggers**	
Defined anxiety	93 (78.2%)
Screen time > 2 h	89 (74.8%)
Junk food consumption	58 (48.7%)
Irregular sleep	50 (42.0%)
**Family history of migraine**	26 (21.8%)
**Treatment**	
Cyproheptadine	66 (55.5%)
Attack	19 (16.0%)
SSRI	34 (28.6%)
**Lifestyle adherence**	88 (73.9%)
**Improvement at the 3rd month**	107 (89.9%)

Descriptive statistics for quantitative data were displayed as * mean ± SD or ** median (min–max) where appropriate. SSRI: selective serotonin receptor reuptake inhibitors. Bold entries in the first column indicate main categories; the rows beneath each bold entry are subgroups.

**Table 3:** The comparisons between groups with and without treatment success at the 3rd month in terms of demographic and clinical characteristics.

	Treatment Failure (n = 12)	Treatment Success (n = 107)	*p*-Value
Age (years) *	8.1 ± 3.7	8.8 ± 4.0	0.542 ^A^
**Gender**			>0.999 ^B^
Female	6 (50.0%)	55 (51.4%)	
Male	6 (50.0%)	52 (48.6%)	
Number of attacks per 6 months **	6 (3–10)	5 (2–15)	0.379 ^C^
Duration (days) **	1.5 (1–7)	1 (1–8)	0.182 ^C^
**Hospitalization**	8 (66.7%)	54 (50.5%)	0.447 ^B^
**Potential triggers**			
Defined anxiety	9 (75.0%)	84 (78.5%)	0.723 ^D^
Screen time > 2 h	6 (50.0%)	83 (77.6%)	0.072 ^D^
Junk food consumption	4 (33.3%)	54 (50.5%)	0.411 ^B^
Irregular sleep	7 (58.3%)	43 (40.2%)	0.369 ^B^
**Family history of migraine**	3 (25.0%)	23 (21.5%)	0.723 ^D^
**Treatment**			**0.024** ^E^
Cyproheptadine	9 (75.0%)	57 (53.3%)	
Attack	3 (25.0%)	16 (15.0%)	
SSRI	0 (0.0%) ^a^	34 (31.8%) ^a^	
**Lifestyle adherence**	4 (33.3%)	84 (78.5%)	**0.002** ^D^

Descriptive statistics for quantitative data were displayed as * mean ± SD or ** median (min-max) where appropriate. ^A^ Student’s *t* test, ^B^ Continuity corrected χ^2^ test, ^C^ Mann–Whitney U test, ^D^ Fisher’s exact test, ^E^ Fisher–Freeman–Halton test. ^a^ The difference between groups was found to be statistically significant (*p* = 0.018). SSRI: selective serotonin receptor uptake inhibitors. Bold entries in the first column indicate main categories; the rows beneath each bold entry are subgroups. Bold values within the table indicate statistically significant differences (*p* < 0.05).

**Table 4:** Comparison of demographic features and treatment success among patients receiving lifestyle modification, cyproheptadine, or SSRI therapy.

	Cyproheptadine (n = 66)	Attack (n = 19)	SSRI (n = 34)	*p*-Value
Age (years) *	6.7 ± 3.0 ^a^	8.5 ± 3.1 ^b^	12.8 ± 2.5 ^a,b^	**<0.001** ^A^
**Gender**				0.226 ^B^
Female	33 (50.0%)	13 (68.4%)	15 (44.1%)	
Male	33 (50.0%)	6 (31.6%)	19 (55.9%)	
Number of attacks per 6 months **	6 (2–15) ^c^	3 (2–8) ^b,c^	8 (2–12) ^b^	**0.007** ^C^
Duration of attacks (days) **	1 (1–7) ^a^	1 (1–3) ^b^	2 (1–8) ^a,b^	**0.002** ^C^
**Hospitalization**	29 (43.9%) ^a^	7 (36.8%) ^b^	26 (76.5%) ^a,b^	**0.003** ^B^
**Potential triggers**				
Defined anxiety	50 (75.8%) ^a^	10 (52.6%) ^b^	34 (100%) ^a,b^	**<0.001** ^B^
Screen time > 2 h	42 (63.6%) ^a^	14 (73.7%) ^b^	33 (97.1%) ^a,b^	**<0.001** ^B^
Junk food consumption	28 (42.4%) ^c^	15 (78.9%) ^b,c^	15 (44.1%) ^b^	**0.016** ^B^
Irregular sleep	39 (59.1%) ^a^	8 (42.1%) ^b^	3 (8.8%) ^a,b^	**<0.001** ^B^
**Lifestyle adherence**	53 (80.3%) ^a^	16 (84.2%)	19 (55.9%) ^a^	**0.017** ^B^
**Treatment success**	57 (86.4%) ^a^	16 (84.2%) ^b^	34 (100.0%) ^a,b^	**0.024** ^D^

Descriptive statistics for quantitative data were displayed as * mean ± SD or ** median (min-max) where appropriate. SSRI: selective serotonin receptor uptake inhibitors. ^A^ One-way ANOVA, ^B^ Pearson’s χ^2^ test, ^C^ Kruskal–Wallis test, ^D^ Fisher–Freeman–Halton test. ^a^ Cyproheptadine vs. SSRI (*p* < 0.05), ^b^ Attack vs. SSRI (*p* < 0.05), ^c^ Cyproheptadine vs. Attack (*p* < 0.05). Bold entries in the first column indicate main categories; the rows beneath each bold entry are subgroups. Bold values within the table indicate statistically significant differences (*p* < 0.05).

**Table 5:** The status of improvement at the 3rd month according to type of treatment and lifestyle adherence.

	Cyproheptadine	Attack	SSRI	*p*-Value ^A^
**Lifestyle nonadherence group**				**<0.001**
Treatment failure	5 (38.5%) ^a^	3 (100.0%) ^b^	0 (0.0%) ^a,b^	
Treatment success	8 (61.5%) ^a^	0 (0.0%) ^b^	15 (100.0%) ^a,b^	
**Lifestyle adherence group**				0.469
Treatment failure	4 (7.5%)	0 (0.0%)	0 (0.0%)	
Treatment success	49 (92.5%)	16 (100.0%)	19 (100.0%)	

^A^ Fisher–Freeman–Halton test. SSRI: selective serotonin receptor uptake inhibitors. ^a^ Cyproheptadine vs. SSRI (*p* = 0.013), ^b^ Attack vs. SSRI (*p* < 0.001). Bold entries in the first column indicate main categories; the rows beneath each bold entry are subgroups. Bold values within the table indicate statistically significant differences (*p* < 0.05).

## Data Availability

All data and documents related to this study are available upon reasonable request from the corresponding author, Cansu Altuntaş (cansu.altuntas@istinye.edu.tr), provided that patient confidentiality is strictly maintained.

## Abbreviations

The following abbreviations are used in this manuscript:

AAM	Acute Attack Management
BMI	Body mass index
CVS	Cyclic vomiting syndrome
EEG	Electroencephalogram
MELAS	Mitochondrial encephalopathy, lactic acidosis, and stroke-like episodes
NSAID	Non-steroidal anti-inflammatory drug
Rome IV	Rome IV Diagnostic Criteria for Functional Gastrointestinal Disorders
SDS	Standard deviation score
SSRI	Selective serotonin reuptake inhibitor
TCA	Tricyclic antidepressant
